# Vascular access: the impact of ultrasonography

**DOI:** 10.1590/S1679-45082016RW3129

**Published:** 2016

**Authors:** Carlos Eduardo Saldanha de Almeida

**Affiliations:** 1 Hospital Israelita Albert Einstein, São Paulo, SP, Brazil.

**Keywords:** Ultrasonoghaphy, Catheterization, central venous, Vascular access devices, Jugular veins, Axillary vein, Subclavian vein

## Abstract

Vascular punctures are often necessary in critically ill patients. They are secure, but not free of complications. Ultrasonography enhances safety of the procedure by decreasing puncture attempts, complications and costs. This study reviews important publications and the puncture technique using ultrasound, bringing part of the experience of the intensive care unit of the *Hospital Israelita Albert Einstein*, São Paulo (SP), Brazil, and discussing issues that should be considered in future studies.

## RESUMO

Punções vasculares são muitas vezes necessárias em pacientes gravemente enfermos. São seguras, mas não isentas de complicações. A ultrassonografia associada à técnica de punção gera diminuição do número de tentativas, de complicações e de custos. O presente artigo revisou importantes publicações sobre o tema, bem como técnicas de punções, trazendo parte da experiência do centro de terapia intensiva de adultos do Hospital Israelita Albert Einstein, em São Paulo (SP) e discutindo tópicos que devem ser melhor explorados em estudos futuros.

## INTRODUCTION

Vascular puncture is a common procedure in intensive care units. This is a fundamental step to access central and peripheral veins as well as for the placement of peripherally inserted central catheters and arterial catheters. It constitutes a critical time in these procedures because many mechanical complications are due to the puncture attempts, including some potentially fatal, such as pneumothorax and hemothorax.

Traditional techniques of vascular puncture that replaced surgical dissection are those guided by anatomical parameters. Such techniques have low incidence of severe mechanical complications that, however, cannot be disregarded. Minor complications, such as arterial puncture (when the target is the adjacent vein), bleeding and local hematoma are not uncommon.^(1)^ Using only anatomical parameters, multiple puncture attempts are often needed, sometimes on different sites, therefore exposing the patients to complications (mechanical and infectious complications), consuming more time to the procedure and causing discomfort to the patient. In addition, the incidence of failure of the vascular catheterization is not irrelevant.^(2)^

For more than three decades, scientific papers have been published suggesting the use of ultrasonography as a imaging method to support vascular puncture.^(3)^ In the last decade, the benefit of this technique was confirmed by systematic reviews and meta-analysis.^(2,4)^ The use of real time ultrasonography, which means that the progression of the needle to the vessel is done under continuous visualization through this imaging method, has many advantages.

This article adresses investigated this issue and determines new fields to be explored using this technique.

### Benefits of the use of ultrasonography for vascular punctures

Hind et al. in a meta-analysis compared central venous catheterization using ultrasound-guided puncture with those carried out guided by anatomical markers. They reported a reduction of 86% in relative risk of catheter placement failures using ultrasonography. In addition, a 57% reduction was seen in relative risk of mechanical complications (p=0.02). The mentioned findings are related to punctures of internal jugular veins. Little can be described about subclavian or femoral vein approaches, because, in the occasion, only one study about each one of these puncture sites was selected for the analysis – both with few studied cases.^(2)^

After 10 years, a new systematic review was published regarding the subject, but at that time, the randomized trials selected were grouped in a meta-analysis regardless of the puncture site. Of 26 papers selected for the review, 19 mentioned internal jugular vein puncture, 3 subclavial veins, 3 femoral veins, 1 approached puncture of internal jugular veins and subclavial veins. Again, benefits of supporting real time ultrasonography were evident. Authors mentioned reduction of 82% of relative risk of fail of catheterization when the equipment is used. The occurrence of arterial punctures, local hematomas, pneumothorax and hemothorax had a reduction of relative risk of 75%, 70%, 79% and 90%, respectively – always with statistical significance.^(4)^

Cost savings follow these advantages. Calvert et al. estimated a saving of 2,000 pounds for each thousand venous central catheterization guided by ultrasonography compared with those guided by anatomic parameters.^(5)^

There is an increase in success rates of catheters placement in the first attempt, both central venous catheters^(2)^ and arterial catheters, in adults and children.^(6)^ A meta-analyses published by Shiloh et al*.* ^(7)^ reported an increase of 71% in the success rate of radial artery catheterizations.

By using ultrasonography, there is also a reduction in the average time spent in the procedure. Despite of the greater time it takes to get materials prepared to be used, as fewer attempts are needed to success, time is saved at the most important part of the procedure: the puncture.^(6)^

### Vascular puncture technique guided by ultrasonography

There is no need of sophisticated ultrasonography resources to perform this procedure. Vascular puncture is can be performed only using the bidimensional mode of the equipment. This is important factor shortens the learning curve of this technique.

It is possible to differentiate vein and artery without use of Doppler effect. To the same pressure done with ultrasound transducer against vessels of the same body segment, the vein collapses before the artery. In addition, the compressed artery tends to be more rounded and it pulses more clearly and in a radial manner.

**Figure 1:**
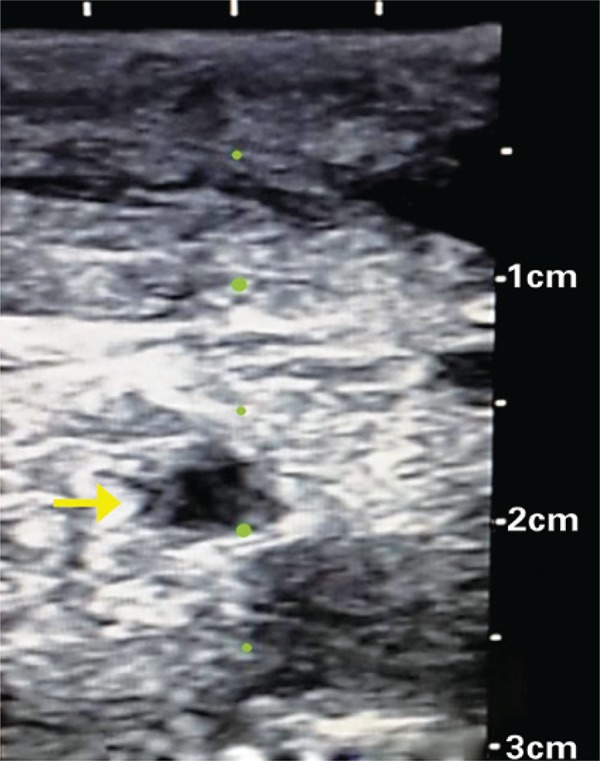
Cross-sectional image of cervical veins showing stenosis of internal left jugular vein (arrow) secondary to previous venous punctures. Note vein with reduced caliber compared with common left carotid artery (bottom to right), as well as thickness of vein wall (dark-grey layer). Compare with figure 2A

Vessels must be evaluated using ultrasonography before the gowning of professional for the procedure. It enables the understanding of their anatomy, identifies possible anatomical variations of the position of the vein in relation to artery, observes the depth of vein to be punctured and recognizes obstacles to a successful procedure, such as thrombosis or stenosis. Venous thrombosis is known by the lack of compressibility of venous segment. In stenosis, a reduction of vein caliber is observed, as well as thickness of its wall (Figure 1). These precautions enable a better planning of the procedure.

**Figure 2:**
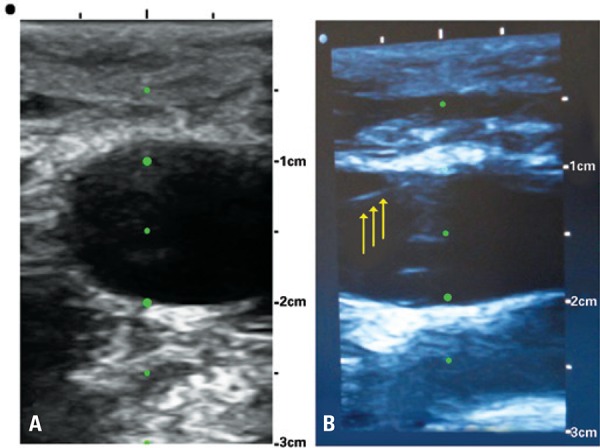
(A) Cross-sectional view. (B) Longitudinal view of internal jugular vein. We observe the presence of guide-wired within the vein (arrows)

Two are the main possibilities of ultrasonography imaging to be used for vascular puncture: one represents a cross-sectional slice of the vessel. (Figure 2A), the other comprises its longitudinal aspect (Figure 2B). Each one has its advantages and disadvantages.

Cross-sectional view of vein to be punctured enables the visualization of adjacent structures. Therefore, it is more evident when needle isguided wrongly to puncture undesired locals, allowing correction of its trajectory. However, the needle is not directly detected on the image until it passes through ultrasonography plan. What is seen, therefore, is the movement of superficial tissues due to distal extremity (bevel) of the needle. It constitutes a good method for beginners because it does not require too much training to keep ultrasonography image sufficiently static for the puncture.

**Figure 3:**
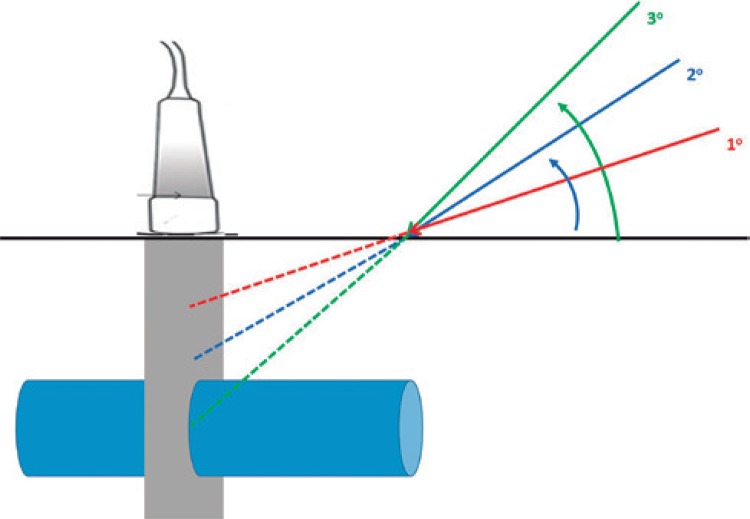
Suggestion of strategy to perform ultrasound-guided vascular puncture using its transverse view

The most rational and didactic form to perform puncture in a vein using ultrasonographic transverse view is to correct the direction of the needle up to the target (vein), as shown in figure 3. Needle is inserted in an angle in relation to the skin surface, in a way that it appears in the image in superficial position in relation to the vessels (red line of figure 3). Normally, the needle is represented on the device screen with a hyperechogenic point followed by posterior acoustic shadow. Once the needle is located, it must be pulled out until it disappears from the ultrasonography image, in order to correct its direction, seeking to match, in the same region, the vein, the ultrasound beam and needle bevel. This process can be repeated as many times as needed until the vessel puncture is achieved (blue and green lines of the figure 3).

Vascular puncture by its longitudinal ultrasound view has the advantage to visualize the entire needle through its trajectory up to the vein. It is the so-called in-plane ultrasound-guided puncture. However, this ultrasonographic view did not show surroundings structures of the target vessels. Therefore, if the prompt visualization of the needle on the ultrasound plan is not achieved, it is possible to perforate or injury unintentionally the structures closed to the target. For this reason, more skill is required to this approach.

**Figure 4:**
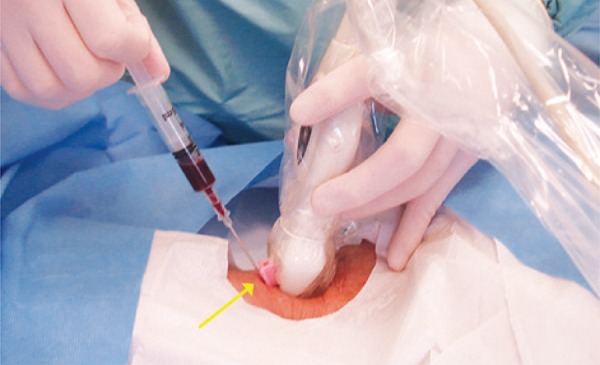
The needle guide (arrow) fixates an insertion angle of the needle in relation to ultrasound transducer

To facilitate the method, needle guides were developed. They are devices that stabilize the angle of the needle insertion in the skin, by fasten the needle to the ultrasound transducer in such a way that needle bevel touches the ultrasound beam in a pre-established depth. The most convenient needle guide must be chosen accordingly to depth of the vein to be punctured (Figure 4).

Regardless of the method chosen for puncture, after aspiration of blood in the syringe connected to the needle (in cases of central venous puncture), or after blood reflow through the needle (in cases of arterial or peripheral veins punctures without syringe), the needle is stabilized in relation to the skin, and the ultrasound transducer is left aside and the insertion of the catheter is done in an usual way (Seldinger technique, catheter-over-needle or catheter within the needle).

### Sites of central venous puncture guided by ultrasonography

There is a high scientific evidence concerning benefit of using ultrasonography in central venous catheterization of internal jugular veins. There is also anatomical reasons for this: (1) in Trendelenburg position these veins often have dilated calibers; (2) generally they are anterior and lateral in relation to common carotid arteries; and, mainly, (3) there are no relevant structures closed to them, with exception of the aforementioned artery.

However, this site is not always available for puncture. For example, in cases that catheters are already placed on or when there is need to change the device to other site. In addition, the catheters inserted by infraclavicular route has less risk of infection compared with those inserted in cervical region.^(1,8)^ In this way, other puncture sites must be better explored in relation to techniques guided by ultrasonography, so they could be used based on better scientific data.

Supraclavicular route for subclavian vein puncture have been widely study in pediatric and neonatal population.^(9,10)^ In last years, studies and case series were published without report complications.^(11,12)^ Image obtained by this route represent, in most of times, the confluence of internal jugular and subclavian veins.

Vascular diameter in this region is used to be greater than veins, separately. Because of accommodation issues of ultrasound transducer in the region, the puncture must be done “in-plane”, as it is done when a longitudinal view of a vessel is used to guide its puncture.

**Figure 5:**
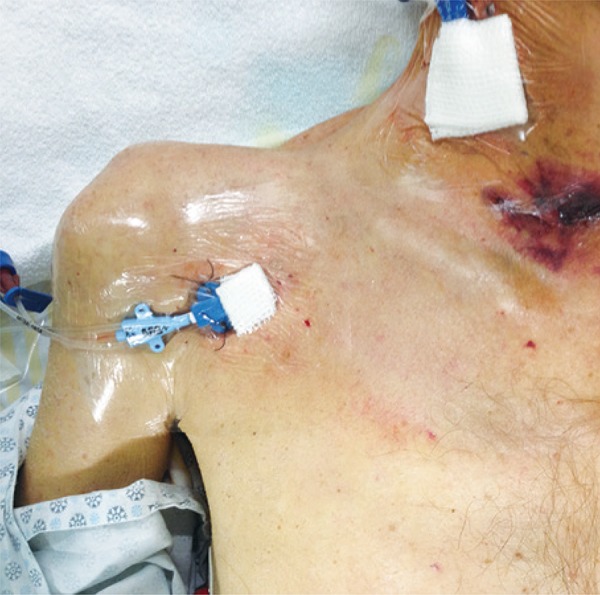
Triple-lumen catheter inserted on right axillar vein with real time ultrasound guidance. It is possible to observe a distance between puncture site and clavicle. There is also a short-term hemodialysis catheter on the right internal jugular vein. The ecchymosis seen in cervical region is due to previous cervicotomy

When vein puncture is made by infraclavicular route using ultrasound guidance, the axillary vein is punctured. This is the name given to these vessels (arteries and veins) when lateral to the first rib. When medial to it, they are called subclavian. Three clinical randomized trials investigated this puncture site.^(13-15)^ They are included in meta-analysis published by Wu et al.^(4)^ Despite the proximity of pleural cavity,^(16)^ the possibility of proceed with the puncture over the rib decrease chances of lung perforation. In addition, this is a compressible site, an useful fact in cases of bleeding due to puncture. However, special attention should be given to identify and avoid injuries to small arterial branches from axillar artery that cross the vein superficially.^(17)^ Other important point to be highlight is that the greater the body mass index of the individual the bigger the depth of axillar veins,^(18)^ which lead to difficulties in the procedure. Figure 5 shows the final positioning and punctured site of a central venous line inserted by puncture of right axillar vein with the support of longitudinal ultrasound view of the vein.

### The experience of Hospital Israelita Albert Einstein

Ten years ago, in the adult intensive care unit of *Hospital Albert Einstein* (HIAE), the use of real time ultrasonography for punctures of central venous accesses become standard. At that time, the physicians that routinely put central venous line received training at institutional training center and, since then, all admitted physicians receive the same training on the beginning of their activities. Based on available literature at the time, it was defined that ultrasonography must be always used for puncture of internal jugular veins and that this site should be the first choice to insert a central venous line. For other puncture sites, the ultrasonography is not mandatory. Therefore, until the present day, in the intensive care unit, internal jugular veins are the most used site for short-term central venous catheters. Of 1,535 central venous line attempts in the adult intensive care unit carried out by on-duty physicians and intensive care residents from January 2012 to January 2015, 1,306 (85.1%) used this site. Of these, only one did not use the ultrasonography to guide the puncture. By using the ultrasonography, more than one puncture was necessary in 302 cases (23.1%) and in 25 (1.9%) there was failure in the catheter placement. This is similar to what is found in the literature.^(4)^ There were no cases of pneumothorax or hemothorax related to these puncture in the analyzed series.

Currently, in the adult intensive care unit of HIAE, the ultrasound-guided puncture is also routinely indicated for puncture of femoral veins and arteries, and radial arteries. Axillary vein puncture is rare in our intensive care unit especially for preferences developed due to the use of ultrasonography throughout the last years. A small group of professionals with affinity to the subject has begun, recently, to implant short-term catheters in axillar veins by infraclavicular route with ultrasound-guided punctures.

### New perspectives: prevention of bleeding complications in central venous punctures

For a long time, the need of hemocomponents transfusion, and more recently the use of hemoderivatives, have been discussed in order to prevent bleeding complications during central venous catheterizations attempts. However, there is no consensus about the real value of this intervention. Nor even is known what is the best laboratorial tests or the best threshold of these tests to indicate this prophylaxis.^(19,20)^

Few studies have evaluated effects of prophylactic use of blood components or blood products in central venous by ultrasound guided punctures. This preventive measures are not free of risks, as it can cause volemic overload or transfusion reactions.^(21,22)^

Without the use of ultrasonography to guide punctures, Fisher et al.^(23)^ reported rate of 9% of minor complications (hematoma formation and bleeding on puncture site) in patients with chronic liver disease and abnormalities of international normalized ratio (INR), activated partial thromboplastin time (APTT) and/or platelet count. There was a case of hemothorax in a patient who had, at the time of venous puncture, INR of 1.5 and 68 thousand platelets/mm^3^.

Gallieni et al.^(24)^ did not find complications in 13 procedures in patients with blood dyscrasias.

In other case series, 133 central venous catheterizations were done using ultrasonography in patients with changes in coagulation, 97% of them in internal jugular veins. Mean count of platelets of patients was 30x10^9^/L, and mean of INR was 3.1. No major complications were reported. Bleeding on puncture site or formation of smaill hematomas was observed in 6% of cases.^(25)^

Della Vigna et al*.* ^(26)^ performed ultrasound guided punctures, most in subclavian veins, in 45 patients with INR, or APTT relation >2.2, and platelets count lower than 50 thousand per mm^3^. There was no complications, excepted in one case in which bleeding on puncture site was solved with local compression.

Literature needs randomized studies on the subject.

The ultrasonography guiding central venous catheterization, which reduces significantly the risk of mechanical complications, associated with the possibility of immediate treatment of bleedings due to coagulopathy using hemoderivatives (spares the preparation that hemocomponents require), raises the idea that prophylactic strategies requiring correction of coagulation tests abnormalities are inadequate. In other words, probably prophylactic hemocomponents transfusions and hemoderivatives infusions prior to central venous punctures are unnecessary, especially when real time ultrasonography is used. However, there are not enough scientific evidence to affirm strongly this statement.

## CONCLUSION

The use of real time ultrasonography reduces complications and vascular puncture cost as well as increases the procedure success rates. Therefore, it should be included in routine practice of these procedures.

There are fields related with subject that still need to be further investigated, such as a better caractherization of the ultrasonography benefits on puncture sites other then internal jugular vein and what role it has in procedures in patients with blood dyscrasias.
